# Unravelling molecular interactions in uracil clusters by XPS measurements assisted by ab initio and tight-binding simulations

**DOI:** 10.1038/s41598-020-69947-3

**Published:** 2020-08-04

**Authors:** Giuseppe Mattioli, Lorenzo Avaldi, Paola Bolognesi, John D. Bozek, Mattea C. Castrovilli, Jacopo Chiarinelli, Alicja Domaracka, Suvasthika Indrajith, Sylvain Maclot, Aleksandar R. Milosavljević, Chiara Nicolafrancesco, Christophe Nicolas, Patrick Rousseau

**Affiliations:** 1 CNR-Istituto di Struttura della Materia, Area della Ricerca di Roma 1, CP 10, Monterotondo Scalo, Italy; 2 Synchrotron SOLEIL, L’Orme de Merisiers, Saint Aubin, BP48, 91192 Gif-sur-Yvette Cedex, France; 3 Normandie Univ., ENSICAEN, UNICAEN, CEA, CNRS, CIMAP, 14000 Caen, France; 4 Biomedical and X-Ray Physics, Department of Applied Physics, AlbaNova University Center, KTH Royal Institute of Technology, 10691 Stockholm, Sweden; 5 Department of Physics, Lund University, P.O. Box 118, 22100 Lund, Sweden

**Keywords:** Chemical physics, Physical chemistry

## Abstract

The C, N and O 1s XPS spectra of uracil clusters in the gas phase have been measured. A new bottom-up approach, which relies on computational simulations starting from the crystallographic structure of uracil, has been adopted to interpret the measured spectra. This approach sheds light on the different molecular interactions (H-bond, π*-stacking*, dispersion interactions) at work in the cluster and provides a good understanding of the observed XPS chemical shifts with respect to the isolated molecule in terms of intramolecular and intermolecular screening occurring after the core–hole ionization. The proposed bottom-up approach, reasonably expensive in terms of computational resources, has been validated by finite-temperature molecular dynamics simulations of clusters composed of up to fifty molecules.

## Introduction

H-bonds and van der Waals interactions are ubiquitous in nature and influence the structure, stability, dynamics, and function of molecules and materials throughout chemistry, biology, physics, and material science. Molecular clusters are weakly bonded systems^1^ with properties different from those of a single molecule or a condensed molecular film. However, the study of the weak interactions in gas-phase clusters of increasing size can give information on structures and mechanisms at work in both the liquid and condensed phases. For instance, gas-phase nucleobase pairs may also follow the Watson–Crick pairing mechanisms^2^. X-ray photoemission spectroscopy (XPS) provides detailed information on the environment of an emitting atom in a sample^3^. In a molecular cluster, the weak, non-covalent interactions modulate the position of nominally equivalent XPS peaks. The measurement and interpretation of such fine variations have provided a better understanding of the chemical equilibrium in ionized aggregates, as well as of their stability and reactivity^4,5^. In the present work the C, N and O 1s XPS spectra of uracil clusters have been measured with synchrotron radiation. The spectra have been interpreted using a simplified bottom-up procedure to describe the interaction patterns within the cluster and a systematic calculation of the ionization energy of all atoms based on ab initio simulations. Different studies on core spectroscopy and fragmentation processes of the isolated uracil molecule have been previously reported^6,7^, and their results provide the reference for the present work.

## Results

**Figure 1:**
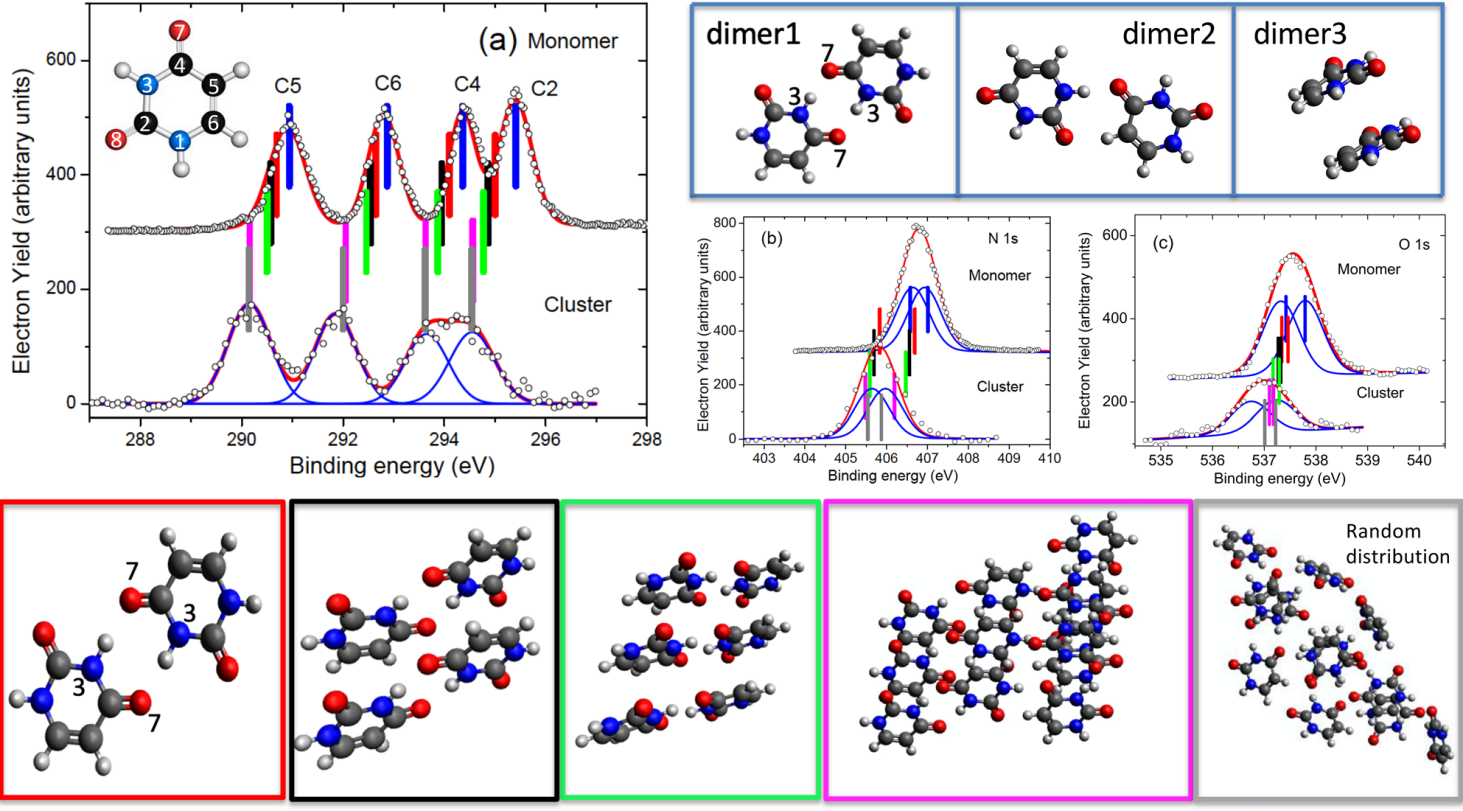
Top panel: the three different structures of the uracil dimers (see text). Central panel (**a**–**c**): the C, N and O 1s XPS spectra ( experiment : open circles; best fit: red full line) of the uracil monomer6 (top) and uracil clusters (bottom) and the average BEs (see Table SI in Supplementary Information) predicted by the DFT theory for the monomer (blue bars), dimer (red bars), tetramer (black bars), hexamer (green bars) and dodecamer (purple bars) uracil clusters, respectively, obtained from the crystallographic structure and for a cluster of 12 molecules obtained by a random distribution (grey bars). Bottom panels: structures of the clusters considered in the calculations.

The XPS spectra of uracil clusters, measured at an oven temperature of 183 ^*◦*^C, are reported in Fig. 1a–c together with those previously measured for the isolated molecule^6^. The uracil ring is formed by two -aza- nitrogen atoms in positions 1 and 3 and four carbon atoms. The C2 and C4 atoms form carbonyl groups with O8 and O7 atoms, respectively. In the isolated molecule (Fig. 1a top), the C1s XPS spectrum displays four distinct features due to the different chemical connections, with the two at higher binding energy (BE) slightly overlapping. The C2 atom has the highest BE because the larger electronegativity of the nearby O and N atoms induces the strongest charge depletion. Vice-versa the peak at the lowest BE has been assigned^6^ to C5, situated in the middle of the conjugated C4-C5-C6 moiety. The two central peaks have been assigned^6^ to C4 and C6, whose shifts depend on the neighboring O and N or only N atom, respectively. All of these assignments have been confirmed by present *ab initio* calculations (see Table SI in Supplementary Information). Each one of the N and O 1s spectra (Fig. 1b,c top), is expected to contain two non-equivalent contributions, with predicted separation of 0.43 eV and 0.37 eV, respectively. In the cluster case, the whole C 1s spectrum is broadened and shifted by about 0.9 eV to lower BE, with the C4 and C2 features clearly overlapping (Fig. 1a bottom). Also the N and O 1s features (Fig. 1b,c bottom) are broadened and shifted to lower BE by a similar amount, within the experimental uncertainty.

## Discussion

To understand how the different interactions in the cluster affect the observed shift a simplified bottom-up theoretical approach has been developed. This approach is motivated by the un-sustainable computational cost of the calculation of XPS lines in large (>10 molecules) clusters, performed on several configurations sampled along equilibrated molecular dynamics trajectories. *A posteriori* we will demonstrate that such approach well reproduces all the short-range structural motifs and intermolecular patterns of realistic clusters. In detail, several different cluster sizes (from dimer to dodecamer) have been cut out from the uracil crystal structure^8,9^ in order to sample all kinds of local connectivity present in the periodic structure, and fully optimized in gas phase, with the BE of each atom calculated, as reported in Table SI of Supplementary Information. As for the connectivity, three different patterns of intermolecular interactions, leading to three possible neutral dimer configurations shown in the top panel of Fig. 1, have been identified: a bidirectional and symmetric H-bond (formation energy per molecule 0.30 eV, *“dimer1”* in Table SII of Supplementary Information), a monodirectional and asymmetric H-bond (0.30 eV, *“dimer2”*) and a stacked dimer (0.06 eV, *“dimer3”*). The formation of large clusters is driven by the anisotropic distribution of H-bond donor and acceptor sites, which can be also modulated by weaker dispersion forces between the *π*-conjugate charge distributions. Calculated average values of the C, N and O1s BEs are shown in Fig. 1a–c, respectively. An overall very good agreement, with slight discrepancies in the case of O1s, is already found for a cluster of 12 molecules, which is a sensible limit for this kind of calculation.

The two different dimer configurations having the same formation energy per molecule, have been simulated first. In both dimers and for all atoms, the BEs shift towards lower values, with a maximum shift of about 1.1 eV for N3 (“dimer1”). This immediately remarks a characteristic and expected property of NH groups H-bonded to electron-rich C=O groups, where the core-hole in the N atom is very efficiently screened by the carbonyl group. This is not the case for N1, which experiences instead a slight increase with respect to the monomer. As for "dimer3" the absence of H-bonds leads to BE shifts ≤ 50 meV (see Table SI of Supplementary). Knowing that the H bond can be partially seen as a resonance of N–H⋯O=C with N^−^⋯H–O^+^–C, we have also checked if it did not lead to an increase of charge on N3 already in the neutral dimer. The results of such investigation, described in detail in the Supplementary Information S2, show that the formation of C=O⋯H–N “prepares” the system, inducing the polarization of the involved atoms without displacing significant amounts of charge between atomic sites. The formation of the core hole takes advantage of the prepared path to provide a more efficient screening of the core holes with respect to the isolated uracil molecule, with the strong polarization of the bond towards the O side playing a key role.

**Figure 2:**
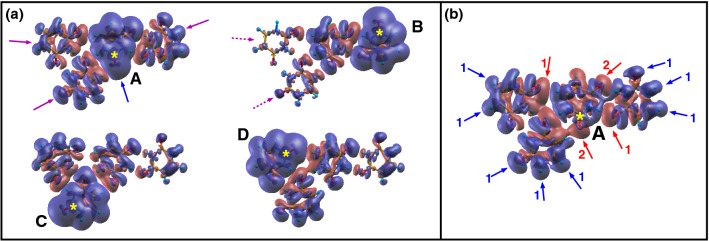
Charge difference-density plots with a sampling of the charge accumulation and depletion regions of 0.0002 a.u./bohr^3^. (**a**) Representation of the overall screening of the core hole (yellow star) generated in the C2 atom in each of the four molecules (A–D) of the tetramer. The full and dashed arrows indicate the contribution of the first and second neighbor molecules. (**b**) Representation of the screening of the core hole in the C2 atom of molecule A due to intermolecular interactions (see text).

Tetramer and hexamer structures have been obtained by piling up two or three *“dimer1”* structures, respectively. In this arrangement the noncovalent, attractive π*-stacking* interactions between the aromatic rings introduce additional collective shifts towards lower BE with respect to the dimer, with an average of 140 meV for the tetramer and a further 100 meV for the hexamer. Such shifts are due to the isotropic participation of neighboring molecules to the screening of the core hole. A tetramer in planar configuration, where the molecules are only connected through H-bonds, has been considered too. This structure implies a higher formation energy per molecule (0.48 eV) with respect to the stacked configuration (0.37 eV). Such a stable planar motif, likely ubiquitous in realistic aggregates, will be discussed below for the interpretation of the intermolecular screening. Finally, the additional shift in the dodecamer with respect to the hexamer varies between 400 meV (C6) and 70 meV (O7), with an average of 220 meV. All calculated C1s BEs are in excellent agreement with the experiments. The shifts in this case depend on the size of the cluster, but not on the connectivity between molecules, because C 1s atoms are not involved in the formation of H-bonds. We note that our “crystallographic structure” model likely tends to overestimate the energy separation between N3 (always involved in bidirectional H-bonds) and N1 (partially involved in monodirectional H-bonds) values and to underestimate the one between O7 and O8. On the basis of the overall satisfactory agreement between calculated and measured BEs, and considering that all the intermolecular and side interactions among the three stacks of uracil molecules (Fig. 2) are active, the dodecamer can be considered as a *“representative”* model of our cluster distribution. To shed light on the intra- and intermolecular contributions to the observed shifts, calculated charge difference-density maps (Fig. 2), which quantify the displacement of the valence electronic charge density from *blue* to *red* zones of the cluster to screen the core hole, have been used.

As an example the ionization of the C2 atom in each one of the four molecules (A to D) of the tetramer in planar configuration has been chosen, due to the abundance of the different intermolecular configurations and a relatively simple graphical representation of this cluster. Two kinds of map have been calculated: in the first case (Fig. 2a) the charge density of the whole cluster containing the core-ionized molecule is subtracted from the charge density of the neutral system. One can clearly see that: (i) the major contribution to the screening of the core hole comes from an intramolecular contraction of the charge density (blue arrow); (ii) the surrounding molecules participate to the screening with an isotropic polarization (magenta arrows) around the hole, with the first neighbors (full arrows) more involved than the second neighboring molecules (dashed arrows). This type of plot clearly shows a cooperative charge redistribution in the cluster, but does not explain the reason why the four C2 atoms in the cluster have different BEs, ranging from 294.35 to 295.37 eV for A and D molecules, respectively. This effect is attributed to intermolecular interactions. To illustrate this and to filter out the intramolecular contribution to the screening, the map in Fig. 2b shows the difference between the electron density of the core-ionized tetramer (in molecule A) and the electron density of the core-ionized molecule (A), alone, plus the charge density of the other three molecules (B, C, D) calculated all together in their neutral ground state. In this way the intramolecular contribution to the screening of molecule A as well as the charge displacement due to the intermolecular interactions between molecules B, C and D in their neutral ground state are removed and the map shows the charge displacement induced in molecules B, C and D by the introduction of the core hole in A. The map clearly shows that the screening charge is effectively moved from the peripheral regions of molecules B, C and D. The blue arrows, labeled 1 in Fig. 2b^,^ identify the peripheral regions of the system where the non-ionized molecules suffer a charge depletion, when the core hole is “turned on”. The charge moves towards the regions adjacent to the ionized molecule in an anisotropic manner, mainly populating the regions of diffuse charge around the O atoms of carbonyl groups (red arrows labeled 1). The two carbonyl groups of molecule A (red arrows labeled 2) are directly involved in the accumulation of the charge density to screen more effectively the core hole. The overall result is a red shift of about 1 eV with respect to the isolated molecule. The similar, but slightly more complex evaluation of the intermolecular screening in the case of molecules B, C and D and the extension to the dodecamer are reported in Supplementary Information S3. These maps graphically demonstrate how an atom of the same species in a molecule located in different positions in the cluster is affected by different forces and interactions, and therefore due to the different screening has a different BE.

**Figure 3:**
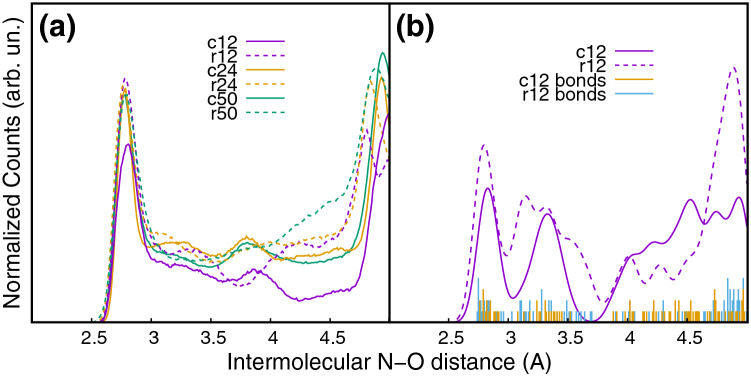
(**a**) Distribution of the (N(H)⋯O) intermolecular distances starting from both the crystallographic structure (full lines) and a random distribution of molecules (dash lines) for clusters of 12, 24 and 50 molecules after 100 ps sampling. (**b**) Distribution of the (N(H)⋯O) intermolecular distances in the case of the most stable structure identified by the metadynamics simulation for a random cluster of 12 molecules with (full line) and without (blue bars) a gaussian convolution (*σ* = 0.1 Å), to account for the thermal distribution, compared with the ones of a cluster of the same size obtained by the crystallographic structure (dashed line and yellow bars).

Finally, we have used finite-temperature molecular dynamics simulations to investigate the connections that can be found in a cluster formed in gas phase by randomly assembling n molecules. The parameter used to evaluate the connectivity in the cluster is the distribution of (N(H)⋯O) intermolecular distances, whose first maximum around 2.8 Å (Fig. 3) describes the H bond. After a benchmark calculation to verify the consistency of the ab initio^10^ and tight-binding^11^ molecular dynamics simulations in the case of the dodecamer, simulations starting from both the crystallographic structure and a random distribution of molecules have been performed for clusters of 12, 24 and 50 molecules. The results are compared in Fig. 3a. In the simulations starting from the crystallographic structure the distribution of the H-bond connectivity is already well defined in a cluster of 12 molecules and, as shown by the intensity of the peak at 2.8 Å, reaches its convergence at 24 molecules. In the case of a random distribution a similar situation is observed, with the convergence already reached for a cluster of 12 molecules. In the case of the dodecamer a metadynamics simulation^12^ for about 200 ps to identify the global energy minimum, has been also performed, following a computational protocol described in detail elsewhere^12^ for the exploration of potential energy surfaces. The most stable structure has been further optimized with the same method (B3LYP) used for the crystallographic structure. The comparison of the connectivity in the crystallographic and randomly oriented clusters (Fig. 3b) shows that the characteristic features of the distribution are clearly present in both simulations, validating the proposed extraction of the cluster structure from the crystallographic one. Then the calculated BEs for the randomly oriented cluster (Fig. 1a–c and Table SI1 in Supplementary Information) are the same of the ones of the crystallographic cluster in the case of C 1s (maximum difference is 50 meV for C6). The splits of the N1s (O1s) lines in randomly oriented clusters reduces (increases) due to the reduction of the site specificity for these atoms, while the disagreement in the absolute value of the O1s BE remains. This latter observation proves that the difference is not due to the definition of the cluster structure.

In summary a theoretical modeling of uracil clusters with a bottom-up approach based on the crystallographic structure has been proposed. Molecular dynamics simulations at finite temperature have confirmed that the model used is substantially able to reproduce connections and structures found in realistic gas phase clusters. Hence this approach can be proposed as a computationally sustainable method to study the chemical physical properties of weakly bonded clusters, provided their crystal structure is known. It can be applied to a large variety of systems from the bases of nucleic acids up to large proteins and therefore find a broad use.

The modeling of the XPS spectra has allowed to disentangle the contribution of the different intermolecular interactions for increasing cluster size and shown the relevance of the H-bonds in the different positions of the cluster to determine the screening of the original core hole. The ability to display the contribution of intra- and inter-molecular variation of the charge density following inner shell ionization provides valuable information on the stability of hydrogen-bonded biomolecules, like for example DNA base pairs in interaction with a damaging radiation. It is indeed known that charge transfer via hydrogen bond, like for example the transport of the protons, can lead to induced and spontaneous mutations^13,14^.

## Methods

### Experiment

The XPS measurements have been performed at the PLEIADES beamline of Soleil synchrotron. A gas aggregation source was used to produce neutral clusters of uracil molecules with a log-normal size distribution centered about 30–50 molecules, depending on the source parameters^15,16^. The details of the experimental procedure are provided in Supplementary Information S4.

## Theory

Ab initio simulations of the properties of uracil clusters, including the calculations of core-ionization energies, have been performed by using the Quantum ESPRESSO suite of programs^17^ in a plane-wave/pseudopotential framework^18–20^. In addition, molecular dynamics simulations to determine the structural properties of clusters at finite temperature have been performed using both ab initio^10^ and tight-binding^11^ methods. As for the metadynamics (MTD), the computational protocol described by Grimme^12^ in his introductory article assessing the functionalities of the xTB program, based on the GFN2-xTB Hamiltonian^11^^,^ for the exploration of potential energy surfaces has been followed. A complete account of all the employed computational protocols is provided in Supplementary Information S1.

## Supplementary information

Supplementary Information
