# Supplementary material for: Unravelling molecular interactions in uracil clusters by XPS measurements assisted by ab initio and tight-binding simulations
Source: Sci Rep. 2020 Aug 4;10:13081. doi: 10.1038/s41598-020-69947-3 (PMC7403593; doi:10.1038/s41598-020-69947-3)
Supplement: Supplementary file 1 — Supplementary Information [file 41598_2020_69947_MOESM1_ESM.pdf]

Supplementary Information

Unravelling molecular interactions in uracil clusters by XPS measurements assisted by ab initio and tight-binding simulations.

Giuseppe Mattioli[1], Lorenzo Avaldi[1], Paola Bolognesi[1], John D. Bozek[2], Mattea C. Castrovilli[1], Jacopo Chiarinelli [1,3], Alicja Domaracka[4], Suvasthika Indrajith[4], Sylvain Maclot [5,6], Aleksandar R. Milosavljević[2], Chiara Nicolafrancesco [2,4], Christophe Nicolas[2] and Patrick Rousseau[4]

[1] CNR-Istituto di Struttura della Materia, Area della Ricerca di Roma 1, CP 10, Monterotondo Scalo, Italy

[2] Synchrotron SOLEIL, L'Orme de Merisiers, 91192 Saint-Aubin, BP48, 1192, Gif-surYvette Cedex, France

[3] Dipartimento di Scienze, Università di Roma Tre, Rome, Italy

[4] Normandie Univ., ENSICAEN, UNICAEN, CEA, CNRS, CIMAP, 14000 Caen, France

[5] Biomedical and X-ray Physics, Department of Applied Physics, AlbaNova University Center, KTH Royal Institute of Technology, SE-10691 Stockholm, Sweden

[6] Department of Physics, Lund University, P.O. Box 118, 22100 Lund, Sweden

S1. Theoretical methods

Ab initio simulations of neutral and core-ionized uracil clusters have been performed by using a plane-wave/pseudopotential/supercell approach, as implemented in the Quantum-ESPRESSO package [1]. The clusters have been cut out from the uracil crystal structure, and equilibrium geometries have been found by fully relaxing all the molecules accommodated in large cubic supercells (up to ~28 Å^3) to minimize the occurrence of spurious interactions between periodically replicated images. Total energies have been calculated using norm-conserving Troullier–Martins atomic pseudopotentials [2], a plane-wave basis set, and the B3LYP hybrid exchange–correlation functional [3]. Satisfactorily converged results have been achieved by using cutoffs of 90 Ry on the plane waves and of 360 Ry on the electronic density, respectively, as well as the Gamma point for the k-point sampling of the Brillouin zone. H(1s), C(2s) and (2p), N(2s) and (2p), O(2s) and (2p) electrons have been treated as valence electrons. All of the inner shell electrons are embedded in the pseudopotentials.

Vertical ionization energies of each atom in each non-equivalent molecule of the clusters have been calculated as total energy differences between ‘‘standard’’ and ‘‘core-hole’’ calculations [4-7]. In the latter case, an excited state pseudopotential containing a 1s core hole has been used in place of the regular pseudopotential, and a different calculation has been performed for each nonequivalent atom. The energy differences between the standard and core-hole calculations have been compared with the corresponding difference obtained for the C (O, N) atom of a CH_4 (CO_2, NH_3) molecule, accommodated in the same supercell, far from the clusters, and used as a reference. Such reference molecules have been chosen because they yield more stable calculations, but practically identical results have been obtained in selected tests performed using the molecules employed as experimental references for chemical shifts. Regarding the calculation of isolated uracil, which is not the target of the present investigation, a further +0.18 eV (+0.34, +0.01) shift has been applied to C 1s (O 1s, N 1s) lines, in order to minimize the mismatch between calculated and measured XPS lines. The same shift has been then applied to all the cluster calculations.

Finite-temperature molecular dynamics, MD, simulations have been performed to investigate connectivity pattern in realistic clusters. As a first benchmark, we have performed ab-initio simulations of a dodecamer by using the ORCA suite of programs [8] in a localized-basis-set framework. In detail, the Kohn–Sham orbitals have been expanded on an all-electron def2-TZVP Gaussian type basis set [9] and the accurate and cost-effective B97-3c composite functional [10], particularly suitable for the investigation of large, non-covalent aggregates. Starting from the dodecamer structure calculated at the B3LYP level of theory discussed above, our MD simulation have been carried out for 10 ps, after 5 ps of thermalization, with the system temperature kept oscillating around 70K by using a Berendsen thermostat. Further, extensive investigation have been performed by using semiempirical MD simulations based on a very recent implementation of the tight-binding method, namely xTB-GFN2 [11], also designed to study geometries, vibrational frequencies and non-covalent interactions (GFN) of large molecular aggregates without system-dependent fitting parameters. In detail, long simulations (50 ps of thermalization followed by 100 ps of sampling) of 12-molecule, 24-molecule and 50-molecule clusters, starting from both crystal structures and random structures (a preliminary 50 ps equilibration at 300K has been also performed in this latter case) have been performed at 70K. A 200 ps meta-dynamics search for global minimum [12] has been finally performed in the case of the 12-molecule cluster. In this latter simulation we have followed the computational protocol described by Grimme [12] in its introductory article assessing the functionalities of the xTB program for the exploration of potential energy

surfaces The method is based on the xTB-GFN2 Hamiltonian [11]. The biasing potential is given as a sum of Gaussian functions and it is expressed with the RMSD of all atoms in Cartesian space as a metric for the collective variables with respect to a starting configuration. More specifically, the biasing potential is expressed as

$$E_{bias}(RMSD) = \sum_{i=1}^{n} k_i \, exp\,(-\alpha\Delta_i)$$

where n is the number of reference structures associated with the pushing ($k_i > 0$) or pulling ($k_i < 0$) strength k, Δ is the collective variable, and the parameter α determines the width (extension in space and time) of the biasing potential. In MTD runs, all the k_i have the same positive values discouraging the system to come back to previous points. The addition of a biasing potential causes the heating of the system, followed by thermalization using the Berendsen thermostat in cycles lasting 1 ps, with extreme acceleration of conformational processes by effectively reducing potential energy barriers. In the case of a 12-uracil cluster, the starting configuration is obtained from an initial random distribution within a sphere (obtained with the Packmol code), and assessed after 50 ps of MD @300K and 150 ps of MD @70 K, as already detailed in the submitted manuscript. We used as specific MTD parameters $k = 0.02$ and $\alpha = 0.5$. We selected the lowest potential energy configuration along the trajectory and re-optimized the structure at the B3LYP-D3 level of theory, before calculating C, N and O XPS lines, finding results very close (within 0.05 eV) to those obtained by simply cutting out the cluster from the crystal structure.

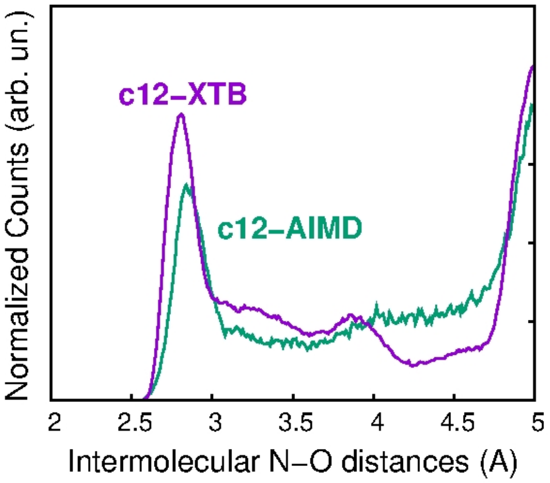

Figure SI1: Distribution of the (N(H)···O) intermolecular distances along molecular dynamics trajectories of a 12-molecule uracil cluster starting from the crystallographic structure, performed at the xTB-GFN2 (c12-XTB purple curve) and ab initio (c12-AIMD green curve) levels of theory.

In order to validate the xTB-GFN2 approach, we have performed a comparative investigation between molecular-dynamics semi-empirical and ab initio simulations, started from the same 12-molecule cluster extracted from the crystal structure and using the parameters discussed above. The results are evaluated by using the same intermolecular N(H)···O distance discussed in the main text for the comparison between clusters composed by a different number of uracil molecules. The main features found in the AIMD plot, including in particular the first peak related to the nearest-neighbor N(H)···O hydrogen bond, are caught by the xTB simulations, with a slight increase in the nearest-neighbor connectivity and a certain degree of structuration of the region between the two main peaks, due to the longer sampling of configurations in the latter case (100 ps vs 10 ps).

Table SI1: The calculated ionization energies of all non-equivalent atoms in each molecule of the cluster. See text for the labeling of the different structures of the molecule in the clusters.

	N1	N3	C2	C4	C5	C6	O7	O8
**Mono (Exp)***	406.58 (406.8)	407.01 (406.8)	295.41 (295.4)	294.36 (294.4)	290.94 (291.0)	292.87 (292.8)	537.42 (537.6)	537.79 (537.6)
**Dimer1**	406.69	405.83	295.00	294.10	290.69	292.65	537.34	537.46
**Dimer2 A**	406.06	406.20	294.94	293.96	290.46	292.35	537.50	537.15
**Dimer2 B**	407.21	406.94	295.65	294.65	290.90	292.93	538.00	538.15
**Ave**	406.63	406.57	295.29	294.30	290.68	292.64	537.75	537.65
**Dimer3**	406.55	406.99	295.41	294.35	290.95	292.88	537.47	537.85
**Tetra1 A**	406.38	405.61	294.69	293.82	290.39	292.37	537.12	537.20
**Tetra1 B**	406.74	405.78	295.02	294.08	290.75	292.71	537.53	537.34
**Ave**	406.56	405.69	294.86	293.95	290.57	292.54	537.32	537.27
**Tetra2 A#**	405.61	405.35	294.35	293.46	289.89	291.66	536.81	536.92
**Tetra2 B**	406.54	405.63	294.82	293.89	290.54	292.47	537.15	537.32
**Tetra2 C**	406.77	406.11	295.10	294.20	290.44	292.50	537.61	537.68
**Tetra2 D**	407.09	406.25	295.37	294.38	291.03	292.86	537.70	537.92
**Ave**	406.50	405.84	294.91	293.98	290.48	292.37	537.32	537.46
**Hexa A**	406.39	405.50	294.68	293.79	290.43	292.40	537.08	537.20
**Hexa B**	406.24	405.49	294.57	293.72	290.27	292.25	537.07	537.10
**Hexa C**	406.77	405.78	295.05	294.10	290.79	292.72	537.38	537.57
**Ave**	406.47	405.59	294.77	293.87	290.50	292.46	537.17	537.29
**Dodeca A**	405.07	404.76	293.77	292.86	289.29	291.10	536.34	536.46
**Dodeca B**	406.03	405.05	294.29	293.39	290.09	292.06	536.75	536.91
**Dodeca C**	406.42	405.73	294.74	293.84	290.06	292.13	537.45	537.37
**Dodeca D**	406.89	405.96	295.13	294.13	290.88	292.68	537.59	537.80
**Dodeca E**	405.24	405.03	293.97	293.09	289.48	291.29	536.60	536.67
**Dodeca F**	406.64	405.58	294.83	293.85	290.64	292.47	537.28	537.49
**Dodeca G**	406.61	405.85	294.89	293.92	290.20	292.22	537.54	537.55
**Dodeca H**	406.71	405.83	294.96	294.00	290.69	292.53	537.62	537.48
**Dodeca I**	405.26	404.88	293.96	293.03	289.50	291.28	536.65	536.48
**Dodeca J**	405.70	404.85	294.00	293.13	289.74	291.73	536.54	536.62
**Dodeca K**	406.47	405.88	294.79	293.93	290.19	292.23	537.61	537.43
**Dodeca L**	407.33	406.40	295.43	294.35	291.16	292.84	538.23	537.94
**Ave**	406.19	405.48	294.56	293.63	290.16	292.05	537.10	537.18
**r12 A$**	405.74	405.43	294.48	293.53	290.00	291.89	536.94	537.18
**r12 B**	405.83	405.61	294.52	293.79	290.30	292.19	537.22	537.09
**r12 C**	406.98	406.37	295.29	294.21	290.76	292.60	537.58	538.03
**r12 D**	405.79	405.58	294.58	293.72	290.27	292.16	537.13	537.26
**r12 E**	405.93	405.60	294.62	293.60	290.02	291.84	537.00	537.37
**r12 F**	406.55	405.60	294.90	293.82	290.43	292.34	537.10	537.56
**r12 G**	405.67	405.35	294.34	293.47	290.04	291.94	536.83	536.95
**r12 H**	405.54	405.07	294.22	293.17	289.76	291.56	536.47	536.98
**r12 I**	405.36	405.05	294.08	293.19	289.65	291.38	536.70	536.83
**r12 J**	405.78	405.78	294.46	293.83	290.26	292.19	537.35	537.03
**r12 K**	405.56	405.38	294.43	293.55	290.13	292.05	536.87	537.10
**r12 L**	405.65	405.61	294.52	293.53	290.00	291.88	536.87	537.26
**Ave**	405.87	405.54	294.54	293.62	290.14	292.00	537.01	537.22

* Ref.[13]
# Tetramer in the planar configuration
$ BEs for a randomly oriented cluster of 12 molecules.

We report in Table SI2 the whole picture of the formation energy per molecule and per dimer (*dimer1*) of all the clusters, discussed in the main text. The values are obtained as differences between the cluster and the isolated-uracil (or dimer) B3LYP+D3 energies, calculated in the same supercell and divided by the number of molecules (dimers) contained in the cluster. The table permits a straightforward comparison of the strength of different kinds of intermolecular connection acting in different clusters. We note that the choice of providing normalized (per molecule or per dimer) values is suitable for the comparison of clusters composed by different amounts of uracil molecules. When comparing with previous literature devoted to uracil dimers, [14,15] the values must be multiplied by 2, showing nice agreement with previous DFT-based and correlated calculations.

Table SI2 : Formation energy of the different structures

	**Formation energy per molecule** (eV)
dimer1 (bidirectional H-bond)	0.30
dimer2 (unidirectional H-bond)	0.30
dimer (stacked molecules)	0.06
tetramer1 (stacked dimers)	0.37
tetramer2 (unidirectional + bidirectional H-bond)	0.48
hexamer (stacked dimers)	0.40
dodekamer (two hexamers connected by unidirectional H-bonds)	0.62
	**Formation energy per dimer** (*dimer1, bidirectional H-bond*) (eV)
tetramer1	0.16
tetramer2	0.37
hexamer	0.22
dodecamer	0.64

S2. Comparison of the increase of the charge on N3 in the neutral cluster due to the H-bond and in the ionic one due to the core hole

The analysis starts from a closer inspection of the charge displacement induced by A) the formation of H-bonds and B) the subsequent introduction of a core hole in an uracil dimer. To

do this, we use two kinds of density difference map, as shown in Figure SI2. The map on the left side has been obtained by subtracting the charge density of the two separated uracil molecules from the charge density of the dimer. This highlights the changes induced by the formation of the two symmetric H bonds. Charge is depleted from blue regions and accumulates in red regions when the intermolecular connection is turned on. On the right side, the second map is obtained by subtracting the charge density of the dimer bearing a core hole at the N3 site indicated by a yellow star from the charge of the ground-state dimer; charge is depleted from blue regions and accumulates in red regions to screen the core hole, whose accumulation region is barely visible because it is buried inside the larger contraction region of diffuse charge around the core hole itself. Both maps are obtained by using the same value of 0.001 a.u./bohr^3 to represent the isosurfaces.

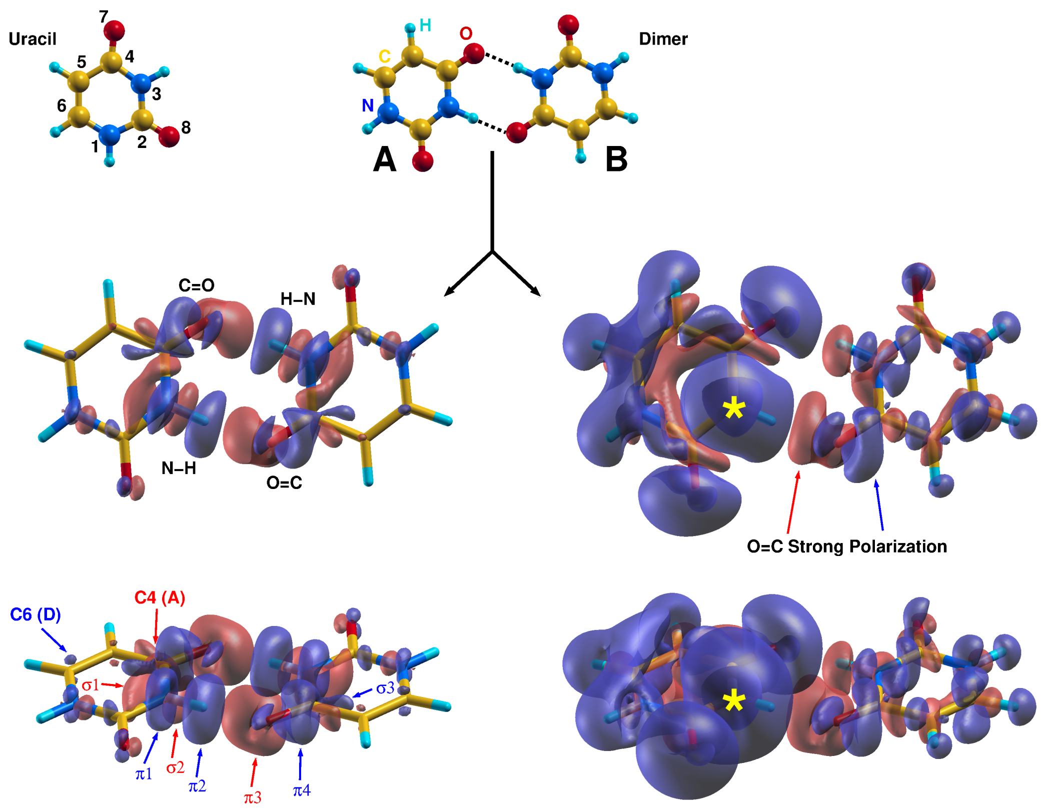

Figure SI2: Difference density maps (see the text for further details) highlighting charge displacements in an uracil dimer following the formation of the dimer (left side, top view above and twisted view below) and the introduction of a core hole in one of the two equivalent N3 positions (left side, top view above and twisted view below). In both maps the isosurfaces of accumulation and depletion are sampled at 0.001 a.u./bohr^3. Optimized geometries of the monomer and of the dimer are shown in the upper part of the figure.

The information extracted from the maps is completed by the calculation of the Bader's charge distribution of O, N and C atoms[16] in ground state and core-hole excited systems. In order to

obtain a well-converged partition of the charge we have doubled the FFT mesh with respect to the standard resolution of DFT calculation implemented in the Quantum ESPRESSO suite of programs.

Table SI3: Bader's charge analysis of the valence charge of an uracil momomer and dimer (see the text). The calculations have been performed in the case of the ground state and of core-hole excited states applied to one of the two symmetric N1 and N3 sites. Atoms involved in the formation of N-H···O=C H-bonds are printed in red, while binding energies of N1 and N3 atoms are printed in green, with the core hole position labeled by a *.

**Bader's charge**	**monomer**			**dimer**		
	ground state	N1*	N3*	ground state	N1*	N3*
**C2-A**	1.35	0.92	0.89	1.35	0.94	0.86
**C4-A**	1.86	1.77	1.60	1.94	1.83	1.58
**C5-A**	3.88	3.84	3.79	3.93	3.81	3.87
**C6-A**	3.09	3.49	3.11	2.99	3.50	3.02
**N1-A**	6.75	**7.87***<br>**406.58**	6.63	6.75	**7.87***<br>**406.69**	6.64
**N3-A**	6.20	6.12	**7.88***<br>**407.01**	6.24	6.21	**7.98***<br>**405.83**
**O7-A**	7.83	7.76	7.83	7.83	7.77	7.83
**O8-A**	7.88	7.89	7.89	7.89	7.90	7.89
**C2-B**	-	-	-	1.35	1.32	1.32
**C4-B**	-	-	-	1.94	1.97	1.99
**C5-B**	-	-	-	3.93	3.92	3.92
**C6-B**	-	-	-	2.99	3.01	3.00
**N1-B**	-	-	-	6.75	6.73	6.73
**N3-B**	-	-	-	6.24	6.26	6.25
**O7-B**	-	-	-	7.83	7.82	7.81
**O8-B**	-	-	-	7.89	7.88	7.88

In the case of an isolated molecule, we can immediately check that atomic charges are compatible with the calculated difference of 0.43 eV between the N1 atom (valence charge = 6.75 a.u., BE = 406.58 eV) and the N3 (valence charge = 6.20 a.u., BE = 407.01 eV). The displacement of valence charge around the core holes is significant in both cases, leading to the very close values of 7.87 a.u. (N1) and 7.88 a.u. (N3) for the Bader's charge of core-ionized N atoms. The formation of the two H bonds induces a spiderweb of displacements

involving π-conjugated charge, partially counteracted by smaller rearrangements of σ charge (we discussed such intricate changes in previous papers [4,6]), highlighted in the left-lower part of the figure. As the sum of accumulation and depletion of charge almost always involves each atom, no very relevant displacement induced by the formation of H bonds is registered in terms of atomic charges (compare the "ground state" columns in Table SI3), but rather a piecewise polarization of the system. We note, in particular, that the most significant net displacement involves the C6 (depletion of around 0.1 a.u.) and C4 (accumulation of around 0.1 a.u.), which can be rationalized as a partial transposition of the C5=C6 double bond, as discussed in detail by Castrovilli et al. [17], in the case of halogenated uracil molecules.

A more significant displacement of valence charge is induced by the formation of core holes in the N1 and N3 positions of the dimer. As discussed in the main text, the core hole is mainly screened by an intramolecular charge displacement, but intermolecular displacements modulate the former, thus providing shifts with respect to pristine XPS N 1s lines up to 1.5 eV in larger clusters. We discriminate here the effect of core-ionization on the N1 position, not involved in the formation of an H bond, compared to the N3 position, involved in the C=O···H-N chain of atoms discussed in detail above. Indeed, the screening of the core hole is more efficient in the N3 position (valence charge = 7.98 a.u., BE shift = -1.18 eV), than in the N1 position (valence charge = 7.87 a.u., BE shift = +0.11 eV), where there is even a tiny blue shift of the BE with respect to the monomer. We can divide such effect in two contributions: 1) As for the intramolecular screening (molecule A in Table SI1), a significant depletion of charge from the C4 atom of molecule A is reported in the case of the N3 core hole only; in other words, the transposition of the C=C bonds related to the formation of the H-Bond provides a surplus of charge that can be used to screen the N3 core hole. In the case of the N1 core hole, the same transposition is reverted back, as indicated by a charge surplus on the C6 atom, but the screening mechanism of the N1 core hole is less efficient because it must counteract the tendency to the transposition toward the C4 position induced by formation of the H-bond. 2) As for the intermolecular screening, there is no appreciable charge transfer from molecule B to molecule A in terms of Bader's charges, as reported in Table SI3. However, we stress the fact that the map reports the charge density difference between a neutral and a core-ionized dimer, that is, the charge displacements due to the formation of the dimer, shown in the left map, are not visible in the right map. Hence, the localization of a core hole on molecule A induces a very strong polarization of molecule B, in particular of the C=O bond highlighted in the figure, thus resulting in a better screening of the core hole in N3.

All in all, there is a twofold effect of C=O groups on XPS lines of uracil cluster. The formation of C=O···H-N "prepares" the system, inducing the polarization of the involved atoms and triggering the transposition of the C5=C6 bond, without displacing significant amounts of charge between atomic sites. The formation of core holes takes advantage of the prepared path to provide, in some cases, a more efficient screening of the core holes with respect to the isolated uracil molecule, with the strong polarization of the bond towards the O side playing a key role.

S3. Evaluation of the intermolecular screening of the core hole

Figure SI3 shows density-difference maps obtained by subtracting from the charge density of a core-ionized system made up by four molecules, the charge density of the same core-ionized molecule (alternatively A, B, C and D), calculated alone, plus the charge density of the remaining three molecules, calculated all together in their ground state. In this map we see neither the intramolecular contribution to the screening of the ionized molecule, nor the charge displacement due to the intermolecular interactions between the remaining three molecules in their ground state. The positive charge regions (in red) show therefore only the areas of the intermolecular screening of the core hole while the negative regions (in blue) the ones where this intermolecular screening comes from. This of course neglects the small intramolecular rearrangements that occur in the target molecule due to the presence of the others. It is quite clear why the molecule A, the most central, is the one with the lowest BE: the charge is effectively moved from the peripheral regions of molecules B, C and D. The blue arrows, labeled 1 in Figure SI3, identify the peripheral regions of the system where the non-ionized molecules suffer a charge depletion when the core hole is "turned on". The charge moves towards the regions adjacent to the ionized molecule in an anisotropic manner, mainly going to populate the regions of diffuse charge around the carbonyl groups (red arrows labeled 1). The two carbonyl groups of the molecule A (red arrows labeled 2) are directly involved in the accumulation of the charge density coming from the periphery of the system, too, and becoming richer in charge they more effectively screen the core hole. The overall result is a red shift of 1.06 eV with respect to the isolated molecule.

Intermolecular screening of the core hole

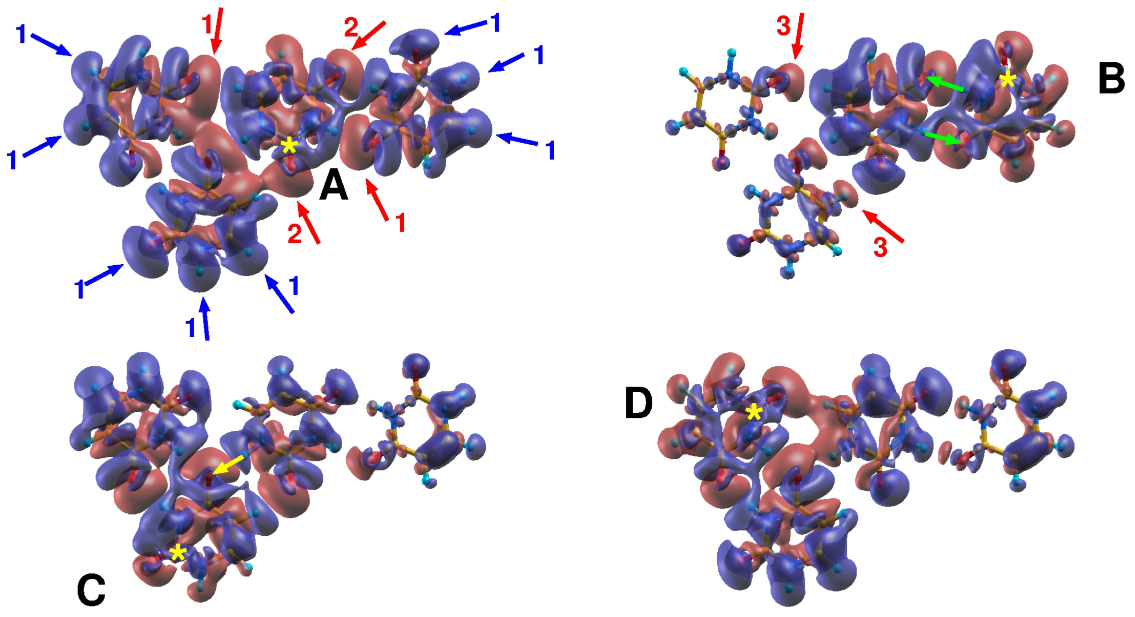

Figure SI3: Charge difference-density plots with a sampling of the charge accumulation (red) and depletion (blue) regions of 0.0002 a.u./bohr[3] in the case of C2 ionization in the four molecules. The blue arrows labelled 1 indicate the contribution from the peripheral region in the case of ionization in molecule A, while the red ones labelled 1 and 2 the accumulation of the charge in the carbonyl regions. The green arrows indicate the role of molecule A and the red ones , labelled 3, of the C and Molecules in the case of ionization of molecule B (see text).

More complex is the evaluation of the larger shield observed in molecule B with respect to the same atom in molecules C and D. A central role is still played by the molecule A, which interacting with B by a bidirectional H-bond (green arrows in Figure SI3) participates to the screen of the core hole recalling charge also from the more distant molecules C and D (red arrows labeled 3). In the case of C and D, on the other hand, the involvement of molecule A in the screening is smaller and the polarization of the molecule C is almost absent. This depends on the unidirectionality of the bond between the group C=O of the molecule C and the NH group of the molecule A (yellow arrows in Figure SI3). This unbalances the system in a dimer A + B, acting as an acceptor with respect to the C + D dimer, acting as a donor.

A similar behavior is also observed in the dodecamer (FigureSI4), where the six peripheral molecules labeled C, D, G, H, K and L are characterized by binding energies on average higher than the others and in some cases even higher than those of the isolated molecule, while the other six molecules have a significantly lower binding energy.

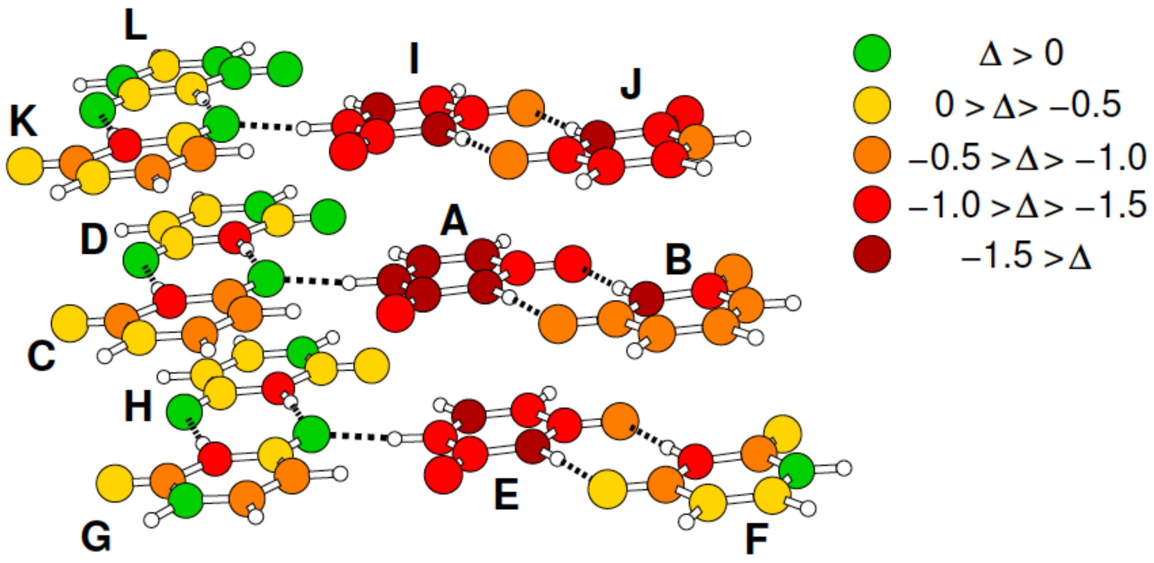

Figure SI4: The structure of the dodecamer cluster and the shifts (represented by the different colors) of the 1s ionization energy for each non-equivalent atom of the twelve molecules with respect to the one of the same atom in the isolated molecule.

S4. Experimental methods

The experiments have been performed at the PLEIADES beamline at the French national synchrotron radiation facility SOLEIL, where a cluster source has been mounted in the dedicate multipurpose source chamber [18] at the beamline.

To produce a beam of neutral molecular clusters a newly designed gas phase aggregation source was used. The design was inspired by a cluster source used in the CIMAP laboratory [19, 20]. The molecular vapor from the commercial uracil power (Sigma Aldrich; purity 99%) was produced by an oven placed in a small chamber filled with helium gas at the pressure of a few mbar. The temperature of the oven was 183°C. Molecules were guided by the helium flow through a condensation channel kept at liquid nitrogen temperature. The residence time of the molecules in the condensation channel is of the order of milliseconds. This time allows to form large clusters via three body collisions in the cold He-buffer gas, leading to the production of clusters with internal energies of about 80 K. Afterwards, the neutral cluster beam is guided to the collision chamber by the helium flow. The cluster beam is directed through a skimmer and crosses the X-ray beam at right angle. Both the exit of the source and the skimmer position can be remotely controlled to align the cluster beam to the photon beam.

The ejected photoelectrons have been detected by a Scienta R4000 electron energy analyzer equipped with a wide-angle entrance lens (±30° aperture). In the present experiment the analyzer has been operated at 200 meV energy resolution.
The C, N and O 1s XPS spectra were recorded at about 60 eV above their respective ionization thresholds, using a pass energy of about 100 eV. The energy spectra were calibrated using a mixture of the molecule under study and a calibration gas with known XPS spectra, i.e. CO_2(C 1s, v = 0 and O 1s) [21, 22] and N_2 (N 1s) [23,21].

References

(1)Giannozzi, P.; Andreussi, O. ; Brumme, T. ; Bunau, O. ; Buongiorno Nardelli, M. ; Calandra, M.; Car, R. ; Cavazzoni, C.; Ceresoli, D.; Cococcioni, M. ; Colonna, N. ; Carnimeo, I.; Dal Corso, A.; de Gironcoli, S.; Delugas, P. ; DiStasio Jr., R. A.; Ferretti, A.; Floris, A.; Fratesi, G.; Fugallo, G.; Gebauer, R. ; Gerstmann, U.; Giustino, F.; Gorni, T.; Jia, J.; Kawamura, M.; Ko, H.-Y. ; Kokalj, A. ; Küçuükbenli, E. ; Lazzeri, M.; Marsili, M. ; Marzari, N. ; Mauri, F.; Nguyen, N. L.; Nguyen, H.-V.; Otero-de-la-Roza, A. ; Paulatto, L. ; Poncè, S.; Rocca, D.; Sabatini, R. ; Santra, B.; Schlipf, M. ; Seitsonen, A. P. ; Smogunov, A. ; Timrov, I.; Thonhauser, T.; Umari, P.; Vast, N.; Wu, X.; Baroni, S. Advanced capabilities for materials with QUANTUM ESPRESSO *J. Phys.: Condens. Matter* **2017**, *29*, 465901. DOI: 10.1088/1361-648X/aa8f79

(2)Troullier, N.; Martins, J. L. Efficient pseudopotentials for plane-wave calculations *Phys. Rev. B*, **1991**, *43*, 1993. DOI: 10.1103/PhysRevB.43.1993

(3)Becke, A. D. Density-functional thermo-chemistry. III. The role of exact exchange *J. Chem. Phys.* **1993**, *98*, 5648. DOI: 10.1063/1.464913

(4)Bolognesi, P.; Mattioli, G.; O'Keeffe, P.; Feyer, V.; Plekan, O.; Ovcharenko, Y.; Prince, K. C.; Coreno, M.; Amore Bonapasta, A.; Avaldi, L. Investigation of Halogenated Pyrimidines by X-ray Photoemission Spectroscopy and Theoretical DFT Methods, *J. Phys. Chem. A* **2009**, *113*, 15136. DOI: 10.1021/jp908512v

(5) Pehlke, E.; Scheffler, M. *Phys. Rev. Lett.* **1993**, *71*, 2338.
DOI:10.1103/PhysRevLett.71.2338

(6) Rondino, F. ; Catone, D.; Mattioli, G.; Amore Bonapasta, A. ; Bolognesi, P. ; Casavola, A.; Coreno, M.; O'Keeffe, P.; Avaldi, L. Competition between electron-donor and electron-acceptor substituents in nitrotoluene isomers: a photoelectron spectroscopy and ab initio investigation *RSC Adv.* **2014**, *4*, 5272. DOI: 10.1039/C3RA45705B

(7) Mattioli, G. ; Filippone, F.; Giannozzi, P.; Caminiti, R.; Bonapasta, A. A. Ab initio Theoretical Investigation of Phthalocyanine–Semiconductor Hybrid Systems *Chem. Mater.* **2009**, *21*, 4555. DOI : 10.1021/cm9014755

(8) Neese, F. The ORCA Program System. *WIREs Comput. Mol. Sci.* **2012**, *2*, 73. DOI : 10.1002/wcms.81; Neese, F. Software update: the ORCA program system, version 4.0. *WIREs Comput. Mol. Sci.* **2017**, *8*, e1327. DOI: 10.1002/wcms.1329

(9) Schäfer, A.; Horn, H.; Ahlrichs, R. Fully optimized contracted Gaussian basis sets for atoms Li to Kr *J. Chem. Phys.* **1992**, *97*, 2571. DOI:10.1063/1.463096 ; Weigend, F.; Ahlrichs, R. Balanced basis sets of split valence, triple zeta valence and quadruple zeta valence quality for H to Rn: Design and assessment of accuracy *Phys. Chem. Chem. Phys.* **2005**, *7*, 3297. DOI: 0.1039/b508541a

(10) Brandenburg, J. G.; Bannwahrt, C.; Hansen, A.; Grimme, S. B97-3c: A revised low-cost variant of the B97-D density functional method J. *Chem. Phys.* **2018**, *148,* 064104. DOI:10.1063/1.5012601

(11) Bannwarth, C.; Ehlert, S.; Grimme, S. GFN2-xTB-An Accurate and Broadly Parametrized Self-Consistent Tight-Binding Quantum Chemical Method with Multipole Electrostatics and Density-Dependent Dispersion Contributions *J. Chem. Theory Comput.* **2019**, *15*, 1652. DOI: 10.1021/acs.jctc.8b01176

(12) Grimme, S. Exploration of Chemical Compound, Conformer, and Reaction Space with Meta-Dynamics Simulations Based on Tight-Binding Quantum Chemical Calculations *J. Chem. Theory Comput.* **2019**, *15*, 2847. DOI: 10.1021/acs.jctc.9b00143

(13) Feyer, V.; Plekan, O.; Richter, R.; Coreno, M.; Vall-llosera, G.; Prince, K. C.; Trofimov, A. B.; Zaytseva, I. L.; Moskovshaya, T. E.; Gromov, E. V.; Schirmer, J. Tautomerism in Cytosine and Uracil: an Experimental and Theoretical Core Level Spectroscopic Study *J. Phys. Chem. A* **2009**, *113*, 5766. DOI:10.1021/jp900998a

(14) Frey, J.A.; Müller; A.; Losada,M.; Leutweyler,S. Isomers of the Uracil Dimer . An ab Initio Benchmark Study *J. Phys. Chem. B* **2007**, *111*, 3535. DOI: 10.1021/jp0683162

(15) Pitonák, M.; Riley,K.E.; Neogràdy,P.; Hobza, P. Highly Accurate CCSD(T) and DFT-SAPT Stabilization Energies of H-Bonded and Stacked Structures of the Uracil Dimer *ChemPhysChem* **2008**, *9*, 1636. DOI: 10.1002/cphc.200800286

(16) Tang,W.; Sanville,E.; Henkelman,G.; A grid-based Bader analysis algorithm without lattice bias. *J. Phys.: Condens. Matter* **2009**, *21*, 084204. DOI: 10.1088/0953-8984/ 21/8/084204

(17) Castrovilli, M. *et al.* An experimental and theoretical investigation of XPS and NEXAFS of 5-halouracils. *Phys. Chem. Chem. Phys.* **20**, 6657–6667, DOI:10.1039/c8cp00026c(2018).

(18) Lindblad, A.; Söderström, J.; Nicolas, C.; Robert, E.; Miron, C. A multipurpose Source Chamber at the PLEIADES beamline at Soleil for Spectrosocopic Studies of Isolated Species : Cold Molecules, Clusters and nanoparticles *Rev. Sci. Instrum.* **2013**, *84*,113105. DOI: 10.1063/1.4829718

(19)Schlathölter, T.; Alvarado, F.; Bari, S.; Lecointre, A.; Hoekstra, R.; Bernigaud, V.; Manil, B.; Rangama, J. ; Huber, B. Ion-Induced Biomolecular Radiation Damage: From Isolated Nucleobases to Nucleobase Clusters *ChemPhysChem* **2006**, *7*, 2339. DOI: 10.1002/cphc.200600361

(20) Holm A. I. S.; Zettergren, H.; Johansson, H. A. B.; Seitz, F.; Rosén, S. ; Schmidt, H. T.; Ławicki, A.; Rangama, J.; Rousseau, P. ; Capron, M.; Maisonny, R.; Adoui, L. ; Méry, A.; Manil, B.; Huber, B. A.; Cederquist H. Ions colliding with cold polycyclic aromatic hydrocarbon clusters *Phys. Rev. Lett.* **2010**, *105*, 213401. DOI : 10.1103/PhysRevLett.105.213401

(21) Hatamoto, T.; Matsumoto, M.; Liu, X.-J.; Ueda, K.; Hoshino, M.; Nakagawa, K.; Tanaka, T.; Tanaka, H.; Ehara, M. ; Tamaki, R. Nakatsuji, H. Vibrationally resolved C and O 1s photoelectron spectra of carbon dioxide *J. Electron Spectrosc. Relat. Phenom.* **2007**, *155*, 54. DOI: 10.1016/j.elspec.2006.10.002

(22) Thomas, T. D.; Shaw, R. W. Jr. Accurate core ionization potentials and photoelectron kinetic energies for light elements *J. Electron Spectrosc. Relat. Phenom.* **1974**, *5*, 1081. DOI: 10.1016/0368-2048(74)85066-8

(23) Kempgens, B.; Kivimaki, A.; Neeb, M.; Köppe, H. M.; Bradshaw, A. M.; Feldhaus, J. A high-resolution N 1s photoionization study of the N2 molecule in the near-threshold region *J. Phys. B: At. Mol. Phys.* **1996**, *29*, 5389. DOI: 10.1088/0953-4075/29/22/016
